# Correlation between Thermodynamic Efficiency and Ecological Cyclicity for Thermodynamic Power Cycles

**DOI:** 10.1371/journal.pone.0051841

**Published:** 2012-12-14

**Authors:** Astrid Layton, John Reap, Bert Bras, Marc Weissburg

**Affiliations:** 1 George W. Woodruff School of Mechanical Engineering, Sustainable Design and Manufacturing, Georgia Institute of Technology, Atlanta, Georgia, United States of America; 2 School of Business and Engineering, Quinnipiac University, Hamden, Connecticut, United States of America; 3 School of Biology, Georgia Institute of Technology, Atlanta, Georgia, United States of America; 4 Center for Biologically Inspired Design, Georgia Institute of Technology, Atlanta, Georgia, United States of America; Dowling College, United States of America

## Abstract

A sustainable global community requires the successful integration of environment and engineering. In the public and private sectors, designing cyclical (“closed loop”) resource networks increasingly appears as a strategy employed to improve resource efficiency and reduce environmental impacts. Patterning industrial networks on ecological ones has been shown to provide significant improvements at multiple levels. Here, we apply the biological metric cyclicity to 28 familiar thermodynamic power cycles of increasing complexity. These cycles, composed of turbines and the like, are scientifically very different from natural ecosystems. Despite this difference, the application results in a positive correlation between the maximum thermal efficiency and the cyclic structure of the cycles. The immediate impact of these findings results in a simple method for comparing cycles to one another, higher cyclicity values pointing to those cycles which have the potential for a higher maximum thermal efficiency. Such a strong correlation has the promise of impacting both natural ecology and engineering thermodynamics and provides a clear motivation to look for more fundamental scientific connections between natural and engineered systems.

## Introduction

### 1.1 Motivation: Ecology and Industrial Networks

A sustainable global community, one that meets the needs of the current generation without sacrificing those of future generations [1] requires the successful integration of environment and engineering. In the public and private sectors, designing cyclical (“closed loop”) resource networks increasingly appears as a strategy employed to improve resource efficiency and reduce environmental impacts [2], [3]. Multiple structural and material flow metrics that one might use to aid in network design exist [4]. These metrics quantify commonsense imperatives to reduce and reuse, but they contain limited, if any, information about sustainable thresholds. Some metrics even hold the potential to mislead [5]. One approach that can improve the efficient use of resources at multiple levels and simultaneously meet sustainable thresholds involves patterning industrial networks on ecological ones [4], [6], [7]. Decades ago, the potential for transferring ecological principles to human systems was recognized as a way to increase the efficient use of energy and resources and reduce waste [8]. In 1989 Frosch and Gallopoulos proposed to convert the traditional manufacturing model, one composed of linear industrial chains of activities, to an integrated model they deemed an ‘Industrial Ecosystem’ [9]. Such a system would use lessons learned from biology to optimize the use of raw materials and energy while minimizing waste through the redefining of effluents as raw material for neighboring processes. Since then, ecological systems have provided analogies for sustainable engineering and industrial systems [4], [7], but there have been few attempts to translate core ecological principles into industrial practice (but cf. [10]). Attempts to organize human systems into more ecologically-realistic patterns continue to be based on the “waste equals food” concept (but cf. [11]) where the output of a given system component (e.g. industry) provides the input for another. While better than previous models, this type of organization does not accurately reproduce the connections patterns of ecosystems where full benefits from the analogy could be realized [6]. In this paper we explore if there are similar advantages for thermodynamic networks.

“To be ultimately sustainable, biological ecosystems have evolved over the long term to be almost completely cyclical in nature, with ‘resources’ and ‘waste’ being undefined, since waste to one component of the system represents resources to another.” – Jelinski, et al. [12]

In 1969, Odum recognized that ecological systems, particularly mature ones, are associated with a high degree of internal recycling of energy and materials, such that the amount of new inputs into the system is small compared to what is transformed among the system components [8]. Human systems in contrast (e.g. agricultural ones) are geared for production rather than efficiency, resembling young rather than mature natural systems. Odum has suggested mimicking mature systems would help shift the focus of human systems from production to efficiency. One desirable property of mature systems is a complex food-web structure; a proliferation of connections between species that exchange material and energy by consuming one another [13]. The extent to which principles derived from ecological systems may be applied in other contexts is unclear. If we can connect the structural properties of ecological networks to well understood physical principles, such as the Laws of Thermodynamics, we might gain sufficient insight to apply ecological lessons to the engineering and development of resource networks [9].

### 1.2 Cyclicity and Thermodynamic Cycles

In this paper we use 28 familiar thermodynamic power cycles of increasing complexity to explore trends in network structure defined by the ecological metric cyclicity [13], [14]. Cyclicity is an older metric reintroduced by Fath and Halnes that measures the presence of cyclic (closed loops as opposed to linear) pathways in a system [13]. Unlike the cycling index (CI), a similar metric which also quantifies the amount of cycling in the system, cyclicity needs no knowledge of flow magnitude, only flow path [8], [15]. Flow magnitude information can be quite complex, if not impossible, to acquire thus cyclicity greatly increases the usefulness and simplicity of the metric as. Cyclicity, which represents what is also known as “strongly connected components” in ecology and graph theory, “refers to the subset of species for which energy can flow from one another and back” [16]. The connections in a system between species, or ‘actors,’ are organized in a matrix form, from which the systems ‘cyclicity’ is calculated. The higher the cyclicity of the system the more interconnected its components. High cyclicity values relate strongly to the overall proportion of the energy retained vs. that which is lost by the system, which may lead to more robust and efficient engineered systems. Fath and Halnes calculated the cyclicity of a number of ecosystems and saw values ranging from 1.62 for the Coachella Valley ecosystem (made up of 30 actors) in Southern California to 14.17 for a mangrove ecosystem with 94 actors [14]. Our results point to the maximum thermal efficiency increasing with cyclicity. So it appears that thermal efficiency, a result of the First Law of Thermodynamics, correlates to a very high degree with an ecological metric based solely on the construction of the system.

Ideal Rankine and Brayton cycles composed the 28 power cycles used. The ideal Brayton cycle is used to model the gas turbine engine and the ideal Rankine cycle is the simplest representation of the vapor power cycles utilized by the electric power generating industry. The inclusions of feedwater heaters, regeneration, reheating and intercooling are all standard ways of increasing the thermal efficiency of the Rankine and Brayton cycles [17]. All of these changes increase the number of times the initial energy in the system is cycled, so it may be reused to reduce the potential heat or work lost and required, thereby decreasing the dependence on outside power. This seems to align with the circuitous structure of food webs favored by nature. As cyclicity is a measure of the existence and *strength* of this internal structural cycling of energy [13], [14], [16] we test if cyclicity can also be used as a measurement tool in thermodynamic power systems, while we explore potential associations with both traditional measures of efficiency and the structure of engineered systems.

## Methods

### 2.1 Conversion to Energy Flow Networks

**Figure 1:**
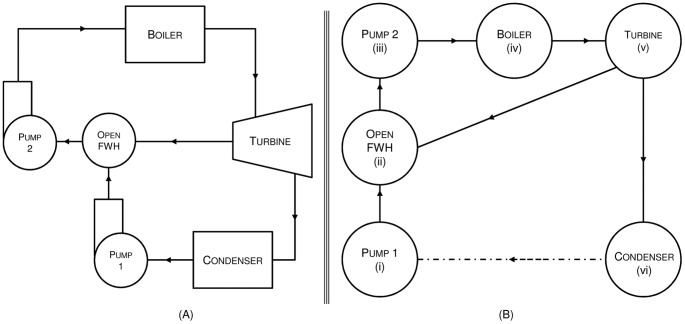
Ideal Rankine power cycle with one open feed water heater redrawn as energy flow networks following thermodynamic network theory [**26**]. Note that the link between the condenser (Node vi) and Pump 1 (Node i) is not a physical flow of energy. Since State 1 acts as an energetic reference state for the network, working fluid returning to that reference state only closes the *material* loop; energy embodied in the working fluid leaving the condenser is rejected to the surroundings.) *(a) Energy, in the form of heat and work and carried by the working fluid, flows to and from the mechanical components of the idealized equipment diagram for a power cycle. (b) The system is simplified with the mechanical components modeled as ‘nodes’ connected by flows of energy in the energy flow diagram.*

To uncover the internal cycling present in the system we must first use the network approach in thermodynamics to construct a graphical model revealing system topology, referred to here as an energy flow network [18]. In this approach mechanical components are considered ‘nodes’ in the network representing the power cycle (a node is a system component that receives and-or transmits energy). Connections between nodes occur when energy embodied in the working fluid as well as internal exchanges of work and heat flow from one node to another. Work and heat entering the cycle from outside are not considered. We analyzed 20 standard variations on the ideal Rankine cycle and 8 standard variations on the ideal Brayton cycle. Only one of the ideal cycles is covered here in detail as the procedure was the same for all cycles used. Figure 1*b* recasts the familiar equipment diagram of an ideal Rankine cycle with one open feedwater heater, seen in Figure 1*a*, as a set of nodes joined by energy exchanges. Starting in the lower left corner of Figure 1*a*, one sees that energy, in the form of shaft work, at Pump 1 enters the system raising the energetic state of the working fluid above that found at State 1 (the reference state for this energy flow network), this translates into the link between node 1 and node 2 in Figure 1*b*. Energy carried by the working fluid flows to the open feedwater heater where it combines with another energy flow in the form of steam bled from the turbine. The network continues the transferring, adding and subtracting of energy as the working fluid moves between ideal components. With the power cycles recast as energy flow networks, we need only to write the structural adjacency matrix and compute its maximum real eigenvalue to determine cyclicity for each cycle.

### 2.2 Structural Matrix

A structural adjacency matrix (**A**), analogous to a connectivity matrix [14], is concerned only with the structural information (links and nodes) of a network and defines the pathways that exist by which material and energy flows from one compartment to another. It is blind to information such as flow rate, quality, and the type of working fluid. A link exists as long as some physical quantity directly joins two nodes (mapped by Figure 1*b*). The adjacency matrix captures flow *direction*. Row space contains information about flow *to* a node, the ‘predator’ in nature, while column space contains information about flow *from* a node, the ‘prey’ in nature.

**Figure 2:**
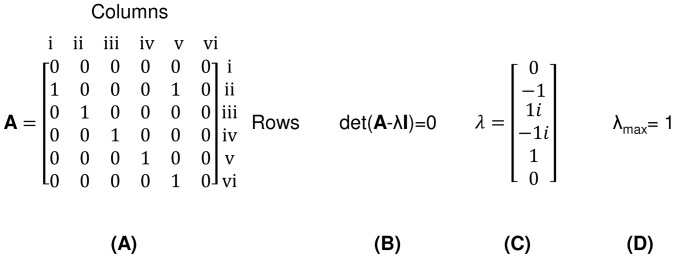
The process for calculating the cyclicity of the 6 component Rankine cycle from Figure 1. (a) Labeled adjacency matrix for the ideal Rankine cycle with one open feed water heater – rows represent flow *to* a node, columns *from* a node. (b) Equation for the calculation of the eigenvalues for the adjacency matrix. (c) Eigenvalues. (d) Maximum real eigenvalue, or the cyclicity, of the cycle.

The adjacency matrix in Figure 2*a* is a structural depiction of the network in Figure 1. The matrix is a binary representation of the connections in the system such that *a_ij_* = 1 if there is a connection from *j* to *i*, and is zero otherwise [14]. For example, energy flowing from Node 1 to Node 2 in Figure 1b is documented by placing a value of 1 in the second row of the first column in the matrix **A** of Figure 2*a*. Flow from Node 2 to Node 3 is indicated by a 1 at [3], [2] and so on.

### 2.3 Maximum Eigenvalue

With the power cycles now in matrix form, cyclicity is found by calculating the maximum real eigenvalue (λ_max_) for each corresponding adjacency matrix. The eigenvalues of a matrix are mathematically defined as the solutions to equation 1: the determinant of the quantity of the matrix in question minus the eigenvalues times the identity matrix of the equivalent size, all equal to zero. The result of equation 1 is a set of eigenvalues (which may be both real and imaginary); MATLAB’s “*eigs*” function was used to execute this task (MATLAB R2011b, Atlanta, Georgia). The maximum real eigenvalue in this set is the cyclicity of matrix **A**, as shown by Borrett et al. [19]. λ_max_ is a measure of the proliferation of pathways that connect two nodes in a network. There is a greater potential for flows to remain within the system as pathways proliferate, λ_max_ is indicative of the resulting internal cycling [17]. The Rankine cycle seen in Figure 1 and represented by the matrix in Figure 2*a* results in a cyclicity value of 1 (λ_max_ = 1) as seen in Figure 2*d*.
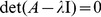
(1)


**Figure 3:**
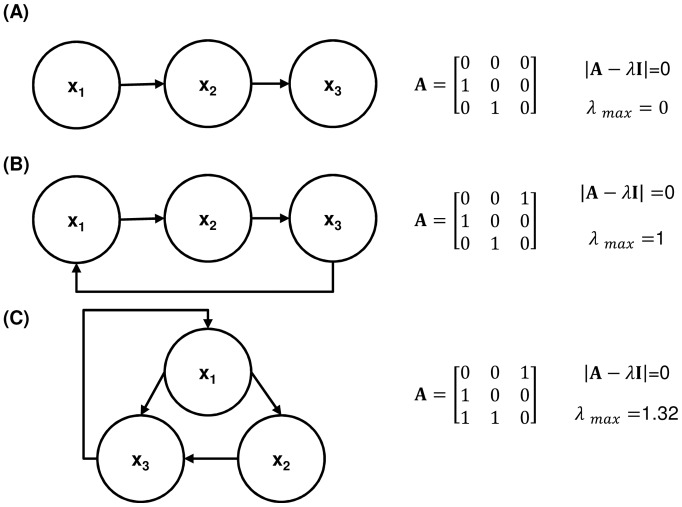
Examples of the three types of internal structural cycling based on cyclicity (eigenvalues). (a) No cycling λ_max_ = 0, (b) weak cycling λ_max_ = 1, (c) and strong cycling λ_max_>1 [10].

Cyclicity can be either 0, 1 or greater than 1. This is illustrated in Figure 3, which is based on the similar figure by Fath and Halnes [14], [20]. Zero cyclicity indicates that no internal cycles are present, Figure 3*a*. Therefore energy traveling through the system never passes through a component twice. A value of one indicates ‘weak cycling,’ meaning only simple closed loop pathways exist, Figure 3*b*. Values of greater than one indicate that the system is made up primarily of complex looped pathways, Figure 3*c*, the larger the cyclicity the more complex and numerous the paths are between components.

The proof presented by Borrett et al. (2007) for the use of eigenvalues to determine the cyclicity (what Borrett et al. call “pathway proliferation rate”) of a system combines results from graph theory and linear algebra [19]. The proof uses the Perron-Frobenius theorem, which guarantees that there is only one real eigenvalue that is greater than or equal to all other eigenvalues (λ_1_≥λ*_i_* for *i* = 2…*n*) in adjacency matrices associated with a network where it is possible to reach every node from every other node (i.e. a strongly connected network) [19]. In strongly connected networks only the maximum (dominant) eigenvalue is left to represent the pathway proliferation rate of the system as the limit of the number of indirect links (pathways between two nodes which consist of more than one link) goes to infinity. Disconnected networks, those networks which have no internal cycling, will have a cyclicity value of zero. Weakly connected networks, those which have cycles made up of one link (self-loops) or have cycling only if link-direction is ignored, may have a maximum eigenvalue of either 1 or 0. [19] Most food webs are composed of networks where large subsets of “nodes” are strongly connected such that the dominant eigenvalue is greater than one, indicating the existence of multiple cyclic pathways.

### 2.4 Thermal Efficiency

All thermal efficiencies (*η_I_* in equation 2) and pertinent state point data were calculated using Engineering Equation Solver (EES) version V8.881-3D. The maximum and minimum cycle temperatures and pressures or pressure ratios were kept constant throughout the modified cycles for consistency, as described in Table 1. Extraction pressures for the feedwater heaters were chosen on a per cycle basis to maximize the thermal efficiency of each cycle. The work and heat externally supplied to the power cycle, W_in_ and Q_in_ respectively, and the work produced by the power cycle, W_out_, were calculated based upon enthalpies (*h*) at pertinent inlet and exit points (outlined by equations 3–5). For more information on calculating work, heat, and the thermal efficiency for thermodynamic power cycles please see a thermodynamic reference book such as Sonntag, Borgnakke, and van Wylen’s *Fundamentals of Thermodynamics* [17].

**Table 1:** Specified state point data for all ideal Rankine and Brayton cycle analyses.

Rankine Cycles - water	Brayton Cycles - air
T_min_ = 318.9 K	T_min_ = 288.2 K
T_max_ = 873.2 K	T_max_ = 1273 K
P_pump1, input_ = 10 kPa	P_compresser, input_ = 100 kPa
P_boiler, input_ = 15000 kPa	r_p_ = 10 (pressure ratio)


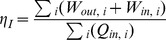
(2)


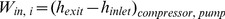
(3)


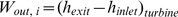
(4)


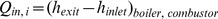
(5)

## Results

Analysis of 28 variations on the ideal Brayton and Rankine cycles shows a positive correlation between cyclicity and the maximum thermal efficiency. The compiled values for cyclicity and thermal efficiency, as well as the specific modifications made to the Brayton and Rankine cycles can be found in Tables 2 and 3. Supporting Figures S1–S6 offer additional insights into the modifications made. The results of these two tables are displayed in Figure 3. The Brayton cycle, by design, gives higher thermal efficiencies than the Rankine cycle, and modifications to the Brayton cycle produce a much larger increase in thermal efficiency than for the Rankine cycle; the addition of one extra component in each (reheat in the Rankine cycle, **R2** in Table 2, and regeneration in the Brayton cycle, **B2** in Table 3) results in a 16.8% increase in thermal efficiency for the Brayton cycle but only a 4.7% increase for the Rankine cycle. Both are desirable, even a small increase in efficiency in practice is highly sought after.

**Table 2:** Thermal efficiency and cyclicity values for 20 (R1–R20) Ideal Rankine power cycles evaluated under the same conditions.

Cycle	ThermalEfficiency (η_I_)	Cyclicity(λ_max_)
**(R1)** Basic Rankine	0.430	0
**(R2)** Rankine with reheat	0.451	1
**(R3)** Rankine with 1 closed FWH trapped condensate	0.453	1
**(R5)** Rankine with 1 open FWH	0.463	1
**(R6)** Rankine with 2 open FWHs	0.472	1.15
**(R8)** Rankine with 1 closed FWH pumped condensate	0.453	1.17
**(R7)** Rankine with 3 open FWHs	0.476	1.21
**(R9)** Rankine with 1 open and 1closed FWH	0.476	1.30
**(R10)** Rankine with 4 open FWHs	0.479	1.24
**(R11)** Rankine with 5 open FWHs	0.480	1.25
**(R12)** Rankine with 6 open FWHs	0.482	1.26
**(R13)** Rankine with 7 open FWHs	0.482	1.27
**(R14)** Rankine with 8 open FWHs	0.483	1.27
**(R15)** Rankine with reheat and 1 open FWH	0.470	1.27
**(R16)** Rankine with reheat and 2 open FWH	0.483	1.33
**(R17)** Rankine with reheat and 3 open FWH	0.488	1.43
**(R18)** Rankine with reheat and 4 open FWH	0.491	1.44
**(R19)** Rankine with reheat and 5 open FWH	0.492	1.45
**(R20)** Rankine with reheat and 6 open FWH	0.493	1.45

* FWH, feed water heater.

**Table 3:** Thermal Efficiency And Cyclicity Values 8 (B1–B8) Ideal Brayton Power Cycles Evaluated Under The Same Conditions [27].

Cycle	ThermalEfficiency (η_I_)	Cyclicity(λ_max_)
**(B1)** Basic Brayton	0.482	1.00
**(B2)** Brayton with Regeneration	0.563	1.22
**(B3)** Brayton with regeneration, intercooling, and reheat (2 turbines)	0.685	1.39
**(B4)** Brayton with regeneration, intercooling, and reheat (3 turbines)	0.718	1.46
**(B5)** Brayton with regeneration, intercooling, and reheat (4 turbines)	0.733	1.50
**(B6)** Brayton with regeneration, intercooling, and reheat (5 turbines)	0.742	1.52
**(B7)** Brayton with regeneration, intercooling, and reheat (6 turbines)	0.748	1.53
**(B8)** Brayton with regeneration, intercooling, and reheat (7 turbines)	0.751	1.54

The vapor power cycles utilized for the generation of 90% of all electric power used throughout the world are modeled by the Rankine cycle [21], [22]. The Brayton cycle is used to model the gas turbine engine. The theoretical upper bound for the efficiency of these and any other real or ideal heat engines is the Carnot efficiency, equation 6. The Carnot efficiency represents the maximum possible work that may be done between any two temperatures and is independent of the working substance used or any particular design feature of the engine. One could continue to increase the number of links added thereby increasing the cyclicity; however, the Carnot efficiency (η_C_) will not be reached. The Carnot efficiency, although physically unattainable, is useful in that it gives us an upper limit to strive for. If the efficiency of a real engine is significantly lower, then additional improvements may be possible. More information on efficiencies and power cycles can be found in any thermodynamic reference book, for example *Fundamentals of Thermodynamics* by Sonntag, Borgnakke, and van Wylen [17]. The Carnot efficiency for the Rankine and Brayton cycles analyzed are 0.635 and 0.774 respectively. We will specify all thermal efficiencies as either maximum Rankine or Brayton cycle efficiencies or Carnot efficiency. The Carnot efficiency creates a ceiling which will lead to a logarithmic-type relationship relating cyclicity to the maximum thermal efficiency if infinite data points were used. Modifications made to real world systems, which must deal with irreversabilities (also known as losses, such as friction), will eventually become cost ineffective in that the addition of feedwater heaters, regeneration, reheating and intercooling will no longer increase cycle efficiency, for example once 8 feedwater heaters are in place in a Rankine cycle [23].
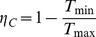
(6)


**Figure 4:**
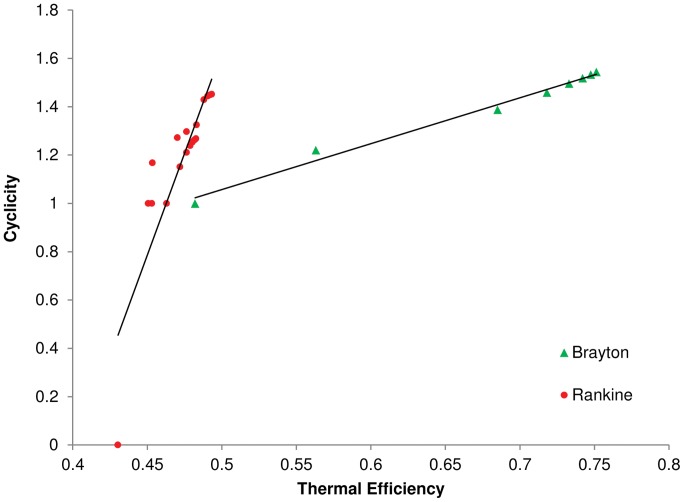
Maximum Thermal Efficiency vs. Cyclicity for all 28 Power Cycles with linear trend lines. Note: All cycles described here are ideal and optimized for maximum thermal efficiency; changes in kinetic and potential energy from one point to another have been neglected as well as losses in connections between components, such as friction losses in pipes, turbulence, and flow separation.

There is a clear lack of data points between the values of zero and one for cyclicity in the Rankine cycles due to the nature of cyclicity being zero, 1, or greater than 1. This constraint makes it impossible to drastically increase the R^2^ value, or coefficient of determination, by obtaining data between the cyclicity values of zero and 1. Including all cycle points (Figure 4) R^2^ values for the linear trend lines are 0.988 and 0.768 for Brayton and Rankine cycles respectively. The R^2^ value, for the Rankine cycle increases to 0.818 if we focus on those cycles which are greater than or equal to one (the Brayton cycles all contain some amount of internal structural cycling and therefore are unaffected by this refocusing).

## Discussion

We conclude from this analysis that the structural method for computing energy cyclicity accurately predicts maximum thermal efficiency for both Rankine and Brayton power cycles. The correlation between cyclicity and maximal thermal efficiency ranges from 0.88 to 0.99, suggesting an extremely strong relationship between these two measures of efficiency. This suggests that increasing the cyclicity (a biological metric) in energetic networks is associated with, or perhaps partially driven by, the maximization of thermodynamic work (an engineering ‘metric’). Alternate power cycle models should be analyzed to further validate the positive relationship between cyclicity and maximum thermal efficiency. From an immediately practical perspective, the benefit of verifying this connection is in determining the relative potential efficiencies of the power cycles. When comparing two modifications to the same cycle it is a great deal easier to calculate cyclicity than to carry out a complete thermodynamic analysis. If cycle A has a higher cyclicity than cycle B, the correlation found here would lead the investigator to believe that cycle A has the potential for a higher maximum thermal efficiency. Establishing this correlation, we can now take advantage of the ecological strategies that we know increase cyclicity, use analogous solutions in human problems, and investigate the extent to which current solutions employing such principles function more effectively.

Our analysis also suggests the two power cycles differ in the extent to which each may be improved by changing the connectivity of its components. The efficiency of the Brayton cycle is extremely sensitive to how interconnected its components are with respect to the transfer of energy. The linear trend lines and coefficients of determination in Figure 4 reveal that less than 2% of the thermal efficiency of a Brayton cycles depends on things other than the internal structural cycling of energy. The thermal efficiency for a Rankine cycle is somewhat less affected by its structural cyclicity, leaving about 23% of the efficiency to depend on other factors. This too may be an area for further study to help clarify the connection for use in engineering design.

Nature’s networks and mankind’s power cycles must both obey the Laws of Thermodynamics, but connecting the two often proves less than straightforward. Although it is well appreciated that thermodynamic constraints affect energy flow in ecological systems [16], ecological systems have been challenging to explain using equilibrium thermodynamic methods. To alleviate this problem, a non-equilibrium perspective is currently in use. This perspective emphasizes the capacity of such complex systems to dissipate energy internally such that they are able to maintain their organization in a physical gradient [24], [25]. Systems with greater structural complexity (such as more mature ecosystems) cycle more energy internally and are associated with stronger physical gradients [24], [25]. Examining power cycles allows us to test the correlation between non-equilibrium and equilibrium thermodynamic measures by computing both cyclicity and thermodynamic efficiency in the same system. The compatibility of both equilibrium and non-equilibrium approaches is shown by the observation that greater cyclicity produced via structural complexity is associated with increases in thermodynamic efficiency.

Finally, our results also suggest additional structural parallels between efficient human vs. natural systems, aside from relationships between structural complexity (number of links) and efficiency. Odum, in his paper *The strategy of ecosystem development* in 1969, observed that the cycling of energy in food webs increases with system maturity, with the bulk of the biological energy flow following detritus pathways [8]. He cites for example a mature forest, where less than 10% of the annual net production is consumed (by grazing) in a living state, most is used as dead matter (detritus) through delayed and complex pathways. Detrital pathways, particularly in mature forests, are composed of low quality energy inputs since the dominant plant biota contain large amounts of relatively refractory structural material. The additional linkages in the modified Brayton and Rankine cycles (**R2**–**R20** and **B2**–**B8**) are put in place to increase thermodynamic efficiency. The added linkages cycle low quality energy (energy entering the system at node 1 is of the highest quality and energy leaving the system is of the lowest quality) through the system, energy which would otherwise be discarded (**R1** and **B1**).

New possibilities and questions appear in the field of industrial ecology and power systems design if the link between cyclicity and thermodynamic efficiency withstands further analysis. Maximization of system work becomes an important goal when aiming to base closed loop industrial systems on ecological ones. One may ask, what is system work in a natural ecosystem? What is the analogy between the average heat input temperature of a thermodynamic power cycle and measurable quantities in an ecosystem? Although answering these answers may or may not yield better system designs, it is doubtful that one would ask the questions were it not for an apparent maximum thermal efficiency-cyclicity correlation. Other analyses will most likely continue to show the importance of cyclical connections to the efficient use and production of energy and matter. Additional cycles, including and beyond thermodynamic ones, should be investigated to broaden the positive relationship seen here to one between any network structure and its efficiency. As the resources that current systems are based on continue to diminish, engineering can only benefit from a greater theoretical structure establishing biology and nature as a source of principles, inspiration and guidance.

## Supporting Information

Figure S1 Basic Rankine cycle idealized equipment diagram for a power cycle (a), energy flow diagram (b). (TIF)

Figure S2 **Rankine cycle with one open feed water heater idealized equipment diagram for a power cycle (a), energy flow diagram (b).**
(TIF)

Figure S3 **Rankine cycle with two open feed water heaters idealized equipment diagram for a power cycle (a), energy flow diagram (b).**
(TIF)

Figure S4 **Basic Brayton cycle idealized equipment diagram for a power cycle (a), energy flow diagram (b).**
(TIF)

Figure S5 **Brayton cycle with regeneration (i.e. counterflow heat exchanger) idealized equipment diagram for a power cycle (a), energy flow diagram (b).**
(TIF)

Figure S6 **Brayton cycle with regeneration (i.e. counterflow heat exchanger), intercooling, and reheat (2 turbines) idealized equipment diagram for a power cycle (a), energy flow diagram (b).**
(TIF)
