# Supplementary figures and images for: Correlation between Thermodynamic Efficiency and Ecological Cyclicity for Thermodynamic Power Cycles
Source: PLoS One. 2012 Dec 14;7(12):e51841. doi: 10.1371/journal.pone.0051841 (PMC3522605; doi:10.1371/journal.pone.0051841)

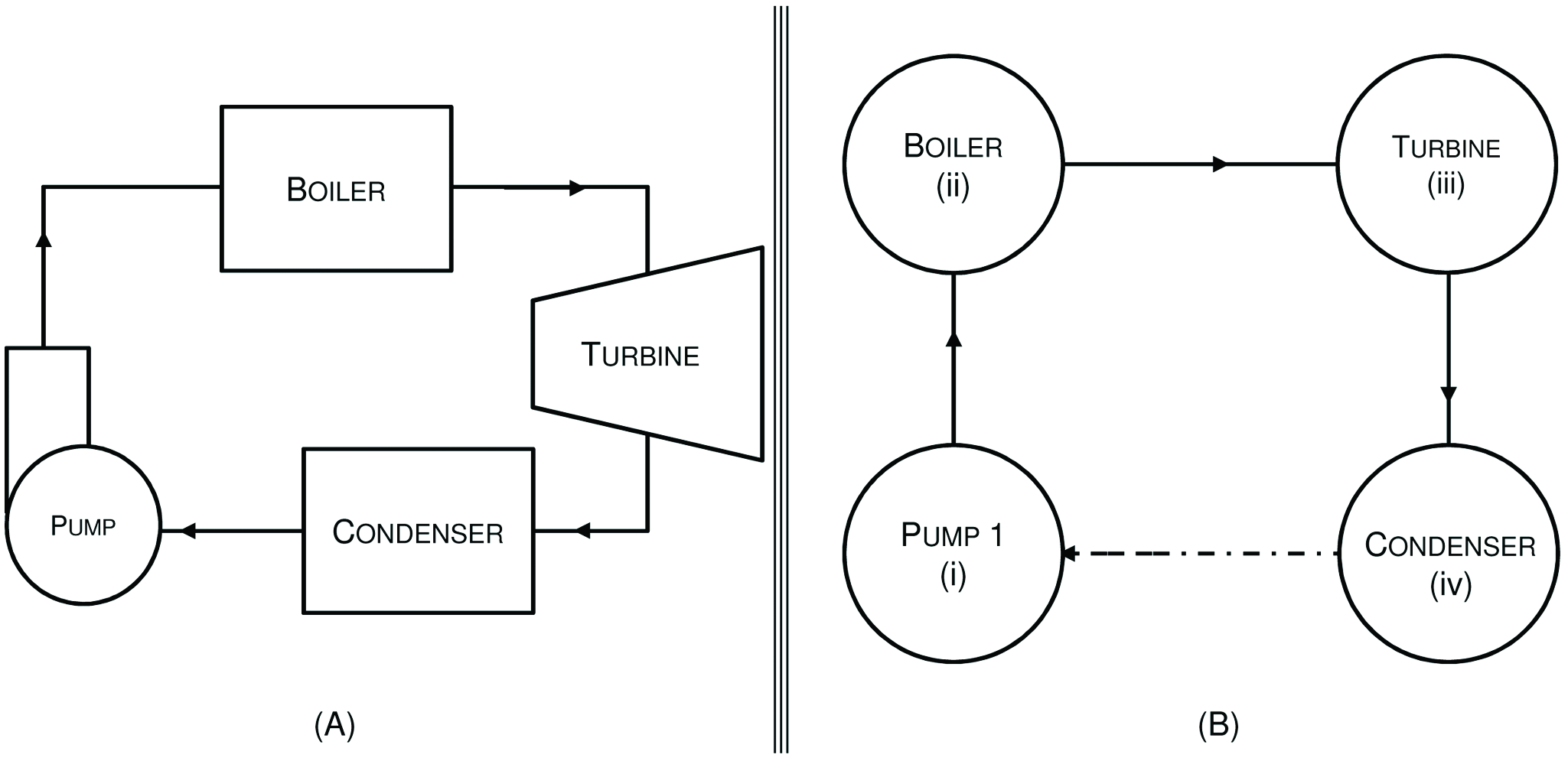

Supplement: Figure S1 — (TIF) [file pone.0051841.s001.tif]

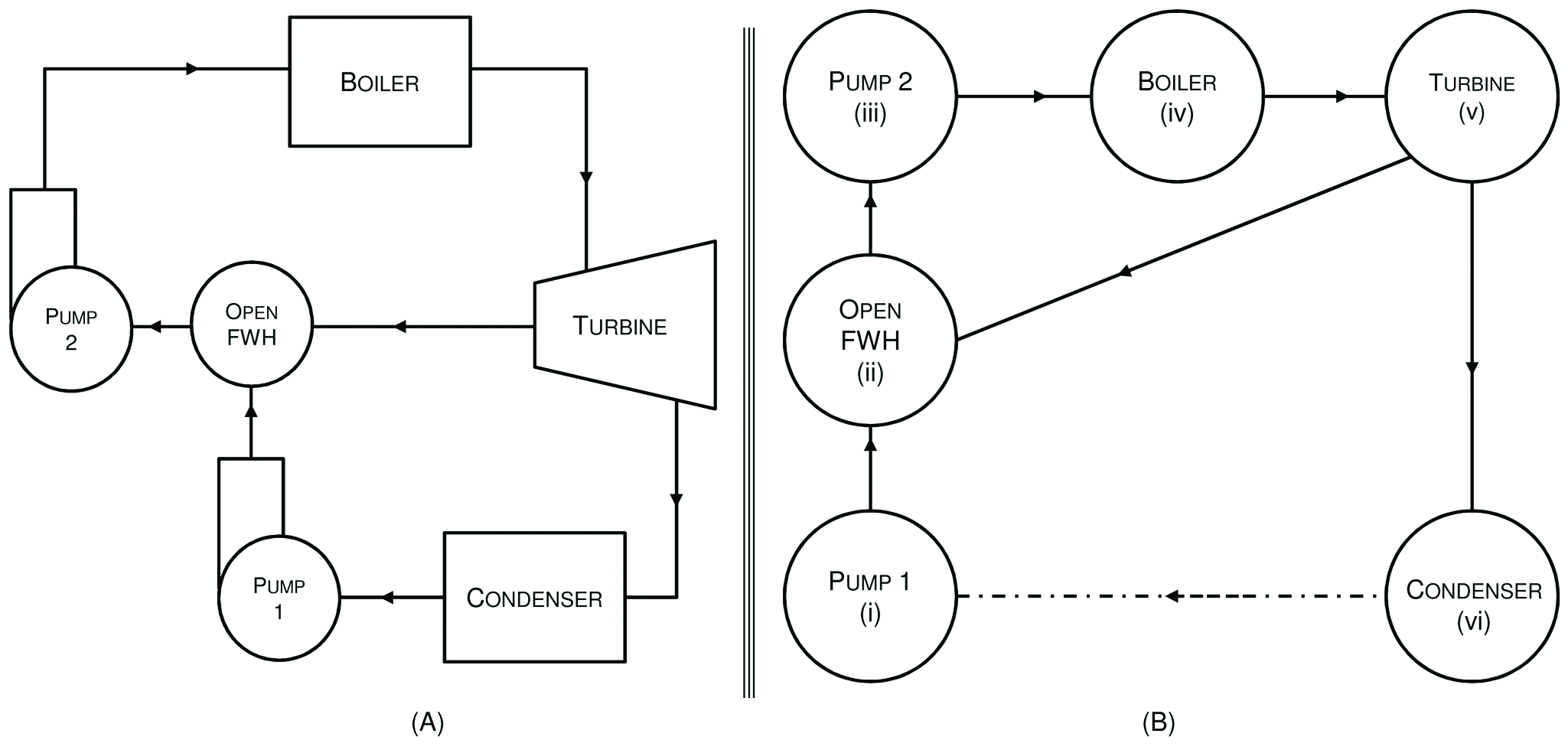

Supplement: Figure S2 — Rankine cycle with one open feed water heater idealized equipment diagram for a power cycle (a), energy flow diagram (b). (TIF) [file pone.0051841.s002.tif]

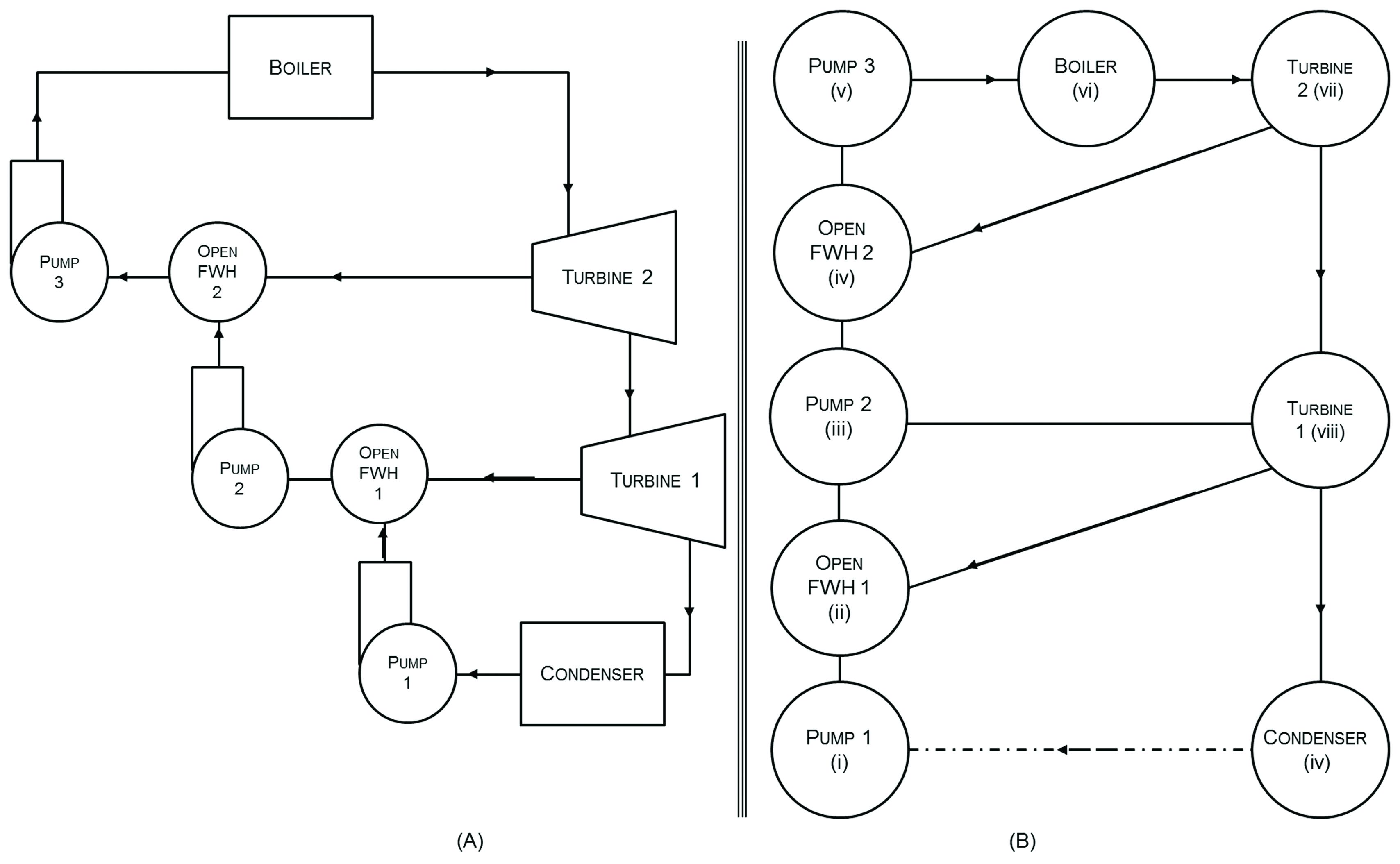

Supplement: Figure S3 — Rankine cycle with two open feed water heaters idealized equipment diagram for a power cycle (a), energy flow diagram (b). (TIF) [file pone.0051841.s003.tif]

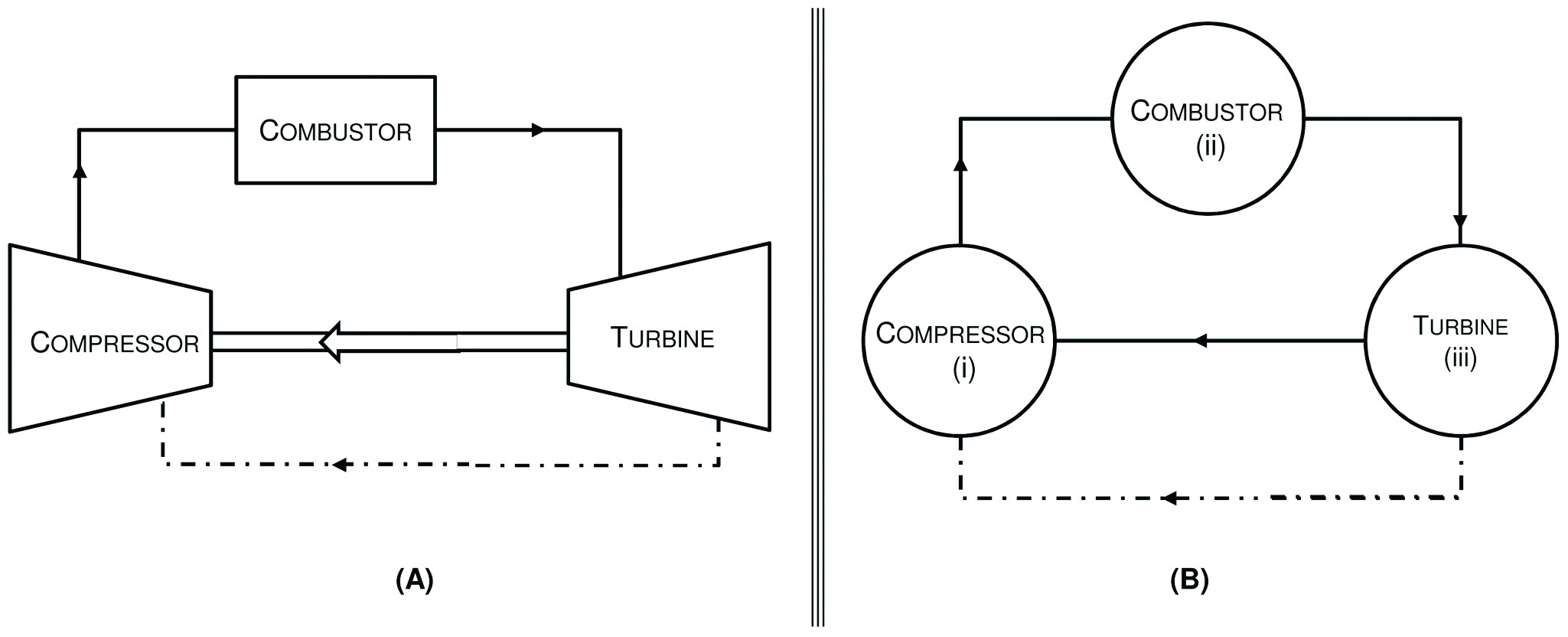

Supplement: Figure S4 — Basic Brayton cycle idealized equipment diagram for a power cycle (a), energy flow diagram (b). (TIF) [file pone.0051841.s004.tif]

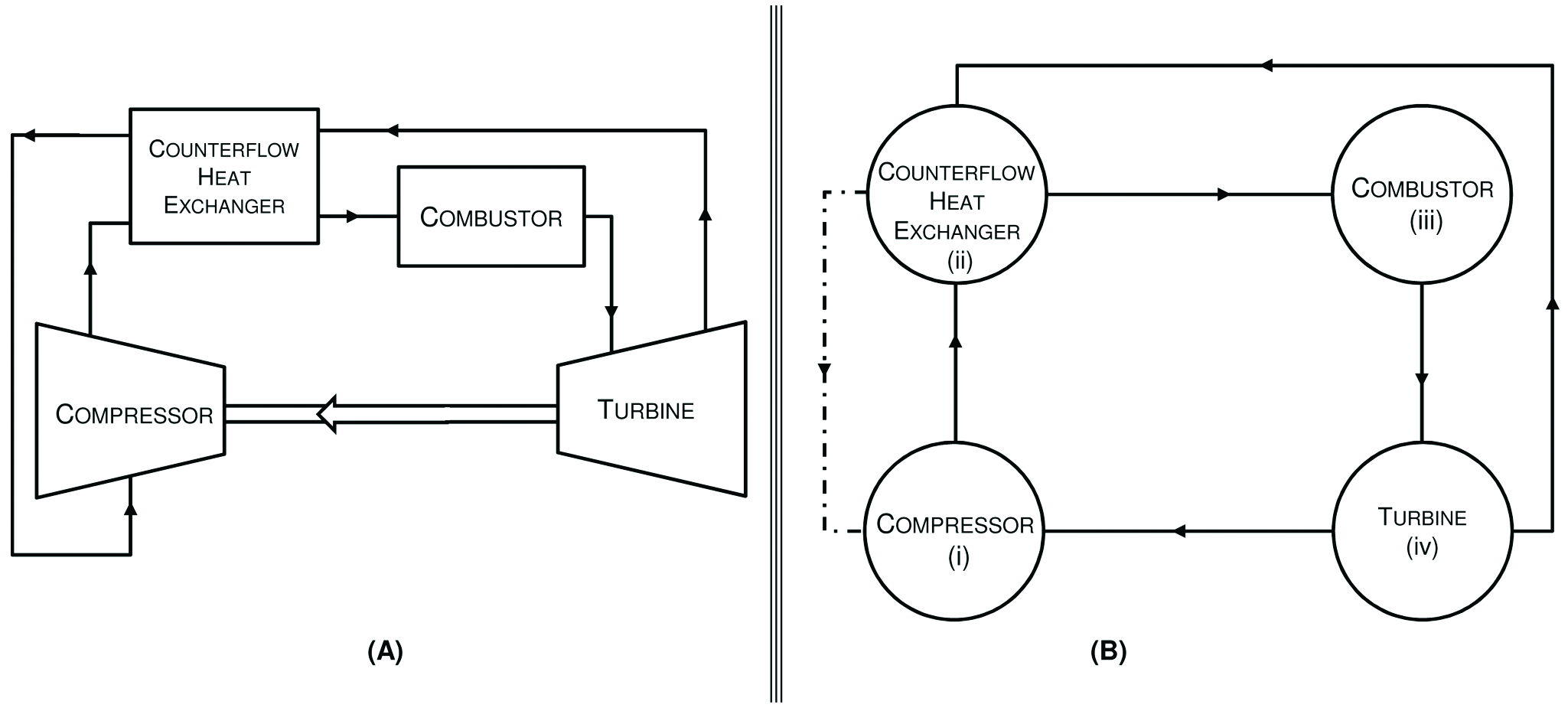

Supplement: Figure S5 — Brayton cycle with regeneration (i.e. counterflow heat exchanger) idealized equipment diagram for a power cycle (a), energy flow diagram (b). (TIF) [file pone.0051841.s005.tif]

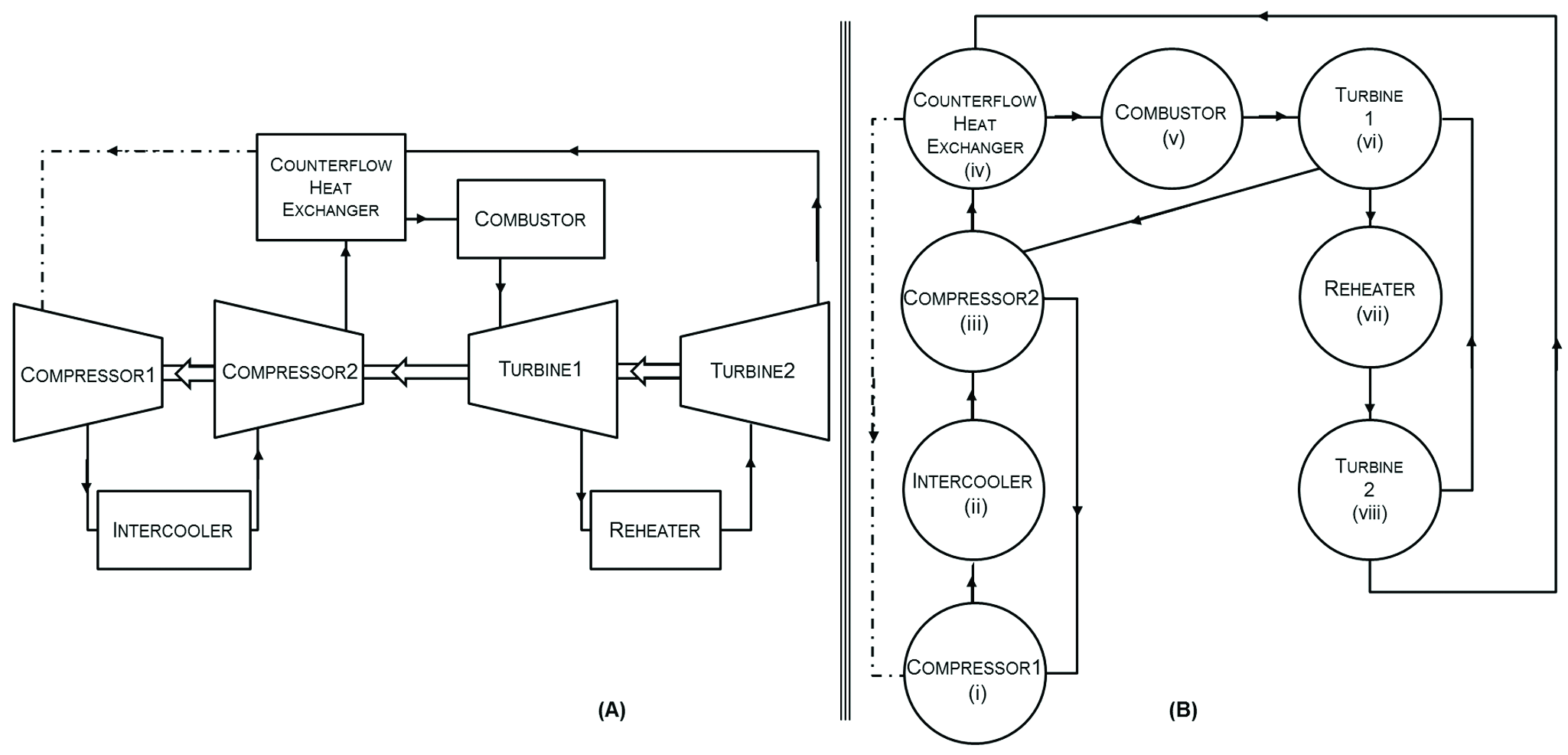

Supplement: Figure S6 — Brayton cycle with regeneration (i.e. counterflow heat exchanger), intercooling, and reheat (2 turbines) idealized equipment diagram for a power cycle (a), energy flow diagram (b). (TIF) [file pone.0051841.s006.tif]
